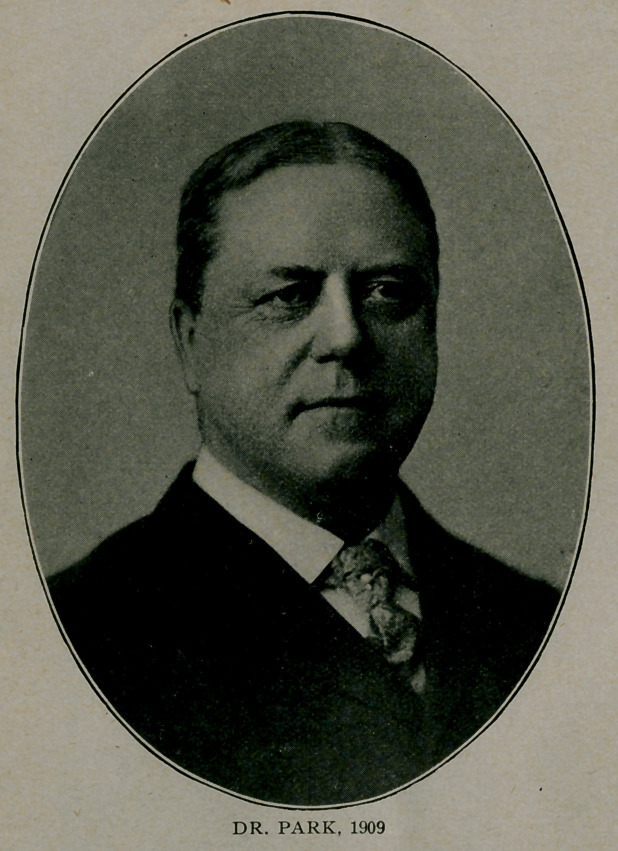# Roswell Park

**Published:** 1914-03

**Authors:** 


### OBITUARIES.

*Readers are requested to report promptly the death of all physicians in Western New York, or former residents of this region, or graduates of any medical school in Western New York and to notify the families of the deceased of our desire to publish adequate obituary notices.*

### Roswell Park

Roswell Park was born May 4, 1852, at Pomfret, Conn., the son of Rev. Roswell Park and Mary Baldwin Park, both of whom were descended from English ancestors who came to America on the Mayflower. His father—the author of several well known hymns—founded Racine College, Wisconsin, and there the son received his baccalaureate degree in 1872, and subsequently the master's degree. In 1876 he was graduated from Rush Medical College in Chicago. In 1880 he married Martha Durkee of Chicago, who died in Buffalo in 1899. Two sons were born of this union, Roswell, Jr., now engaged in business in Buffalo, and Julian, of the faculty of the recently established Arts and Science Course of the University of Buffalo. His sister also survives him.

Dr. Park's professional career may be epitomized as follows:

1877-79—Demonstrator of Anatomy, Women's Medical College, Chicago.

1879-82—Adjunct Prof. of Anatomy, Chicago Medical College.

1883—Called to Buffalo as Prof. of Surgery, University of Buffalo.

1884-93—Surgeon, Fourth Brigade, N. G. N. Y.

Possessed of a logical intellect and a clear and delightful literary style, Dr. Park was well known as an author, his authorship being of that highest type inspired by definite knowledge and the possession of a useful message. Thus, even when the subject was not a technical one and the title might suggest an academic standpoint, his writings always showed a strong prac-

DR. PARK, 1890

tical trend. This fact is well illustrated in the sole posthumous paper (excepting a few accepted for publication before his death) which is published in the present issue of this journal. His bibliographic record is appended, save for some minor contributions to periodic literature and his editorial work in connection with *The Medical Press of Western New York*, published from January, 1886, to June, 1889, when it was incorporated with the BUFFALO MEDICAL JOURNAL.

Dr. Park has been spoken of as a many-sided man, and he sometimes used to refer to himself in jest as a general specialist. Perhaps it would be more correct to say that he showed one concentrated mental proclivity, to base his work on the bedrock of fact, to build broadly and strongly, and that such an intellectual edifice could not but serve many useful purposes. Thus, while a skillful and—for post-anæsthetic days—a rapid operator, he was a surgeon in the broader sense, rather than chirurgeon in the original meaning of the term. He was one of a comparatively few surgeons who did not use knife-blade sights in aiming at a case. For this reason, when he did decide upon operation, his own confidence and that of his associates was greater, and his

DR. PARK, 1901

*Courtesy of Buffalo Express*

attack definite and decisive. While his early experience as a teacher of anatomy was of great value to him as an operator, his research work along lines of pathology and bacteriology, his theoretical and practical grasp of the principles of antiseptic and, subsequently of aseptic surgery, at an early date in their development, were of equal value from the standpoint of technic, they characterized his teaching and authorship and prepared him for future tasks of even greater importance. It was concentration of energy rather than versatility in the ordinary sense that made him a useful member of the Buffalo Society of Natural Sciences and of various other scientific organizations in Chicago and Buffalo, that led the American Association for the

DR. PARK, 1909

Advancement of Science to elect him to the presidency. His early studies logically led to a conception of the necessity of a serious study of cancer and made him the prime mover in the establishment of the New York State Institute for the Study of Malignant Disease. The same logical extension of interests may be seen in his early and persistent studies of radiant energies, although he never posed as a physicist or chemist or devoted himself technically to Roentgenology. His paper published in this issue not only illustrates this interest but exemplifies his grasp of general principles and power to assemble facts, as well as his constant effort toward securing practical humanitarian results from what might appear to the superficial observer as mere scientific abstractions.

Like most other men who have achieved greatness, Dr. Park was a man, a gentleman, a good citizen and a good friend and not merely a highly efficient mental machine. Hence, we find him identified with and honored by various organizations apart from the local and general professional organizations, membership in which may be taken for granted and in which he naturally played a prominent part. He had been president of the Buffalo, Saturn and University Clubs of Buffalo; he was a life member of the Buffalo Fine Arts Academy and of the Buffalo Historical Society; a member of the Park Club of Buffalo, of the University Club of New York, of the Army and Navy Club of Washington. Membership in the last may be considered a professional affiliation, since he was a first lieutenant in the U. S. Medical Reserve Corps and a non-resident professor in the Army Medical School. He was an amateur musician and composer of no mean ability, and exerted a strong influence in the development of musical culture in Buffalo, although too much occupied with other matters to have made this side of his life prominent or even to have made it known to many of his professional associates. Logical incidents in his life, rather than significant landmarks, are the reception of an honorary degree of M. D. from Lake Forest University, of an honorary degree of A. M. from Harvard and of the degree of LL. D. from Yale.

As a teacher, didactic and clinical and, to some degree as a popular lecturer, Dr. Park was clear, interesting and practical. Beginning his career as a medical teacher in the days when medical colleges were still in the evolutionary stage typified by the "little red school house," he was not content merely to fulfill routine duties conscientiously but as one of the most persistent workers toward securing a curriculum, equipment and system of pædagogy in medicine, consistent with the highest standards of education in general. In this respect his influence was more than local. Called to the professorship of surgery in Rush Medical College in 1890, his decision to remain in Buffalo led the profession to tender him a banquet in appreciation of his loyalties to ties so firmly established. This invitation had more than a personal significance. In fact, every personal honor that Dr. Park received was reflected in an altruistic way. The recognition of Dr. Park's attainments came at a time when professional sentiment and legislation were demanding greater things of medical education. The signal honor conferred upon him emphasized to the profession and the public the fundamental worth and the present needs of the University of Buffalo; it gave an additional impetus to the forces already at work within the institution and added to their influence upon the community and, unquestionably, hastened the concrete attainment of means to accomplish the educational ends so long desired.

In 1908, a similar banquet was tendered to Dr. Park, commemorating the completion of a quarter of a century as professor in the University. Dr. Park's attachment to the University of Buffalo was such that it is no coincidence that its welfare was on his mind and the subject of conversation on the evening before his death. His large private medical library is bequeathed to the University of Buffalo and his instruments to the Buffalo General Hospital.

At three o'clock in the morning of Sunday, February 15, Dr. Park was seized with an attack of syncope and died within a few minutes. The funeral was held at Trinity Church, February 17, being attended by representatives of the Council, Faculties and Alumni of the University of Buffalo, of the Buffalo Academy of Medicine and the Medical Society of the County of Erie and of out-of-town organizations, of the Buffalo, Saturn and University Clubs, of the Sons of the Revolution and of various other institutions with which Dr. Park was connected.

## Figures and Tables

**Figure f1:**
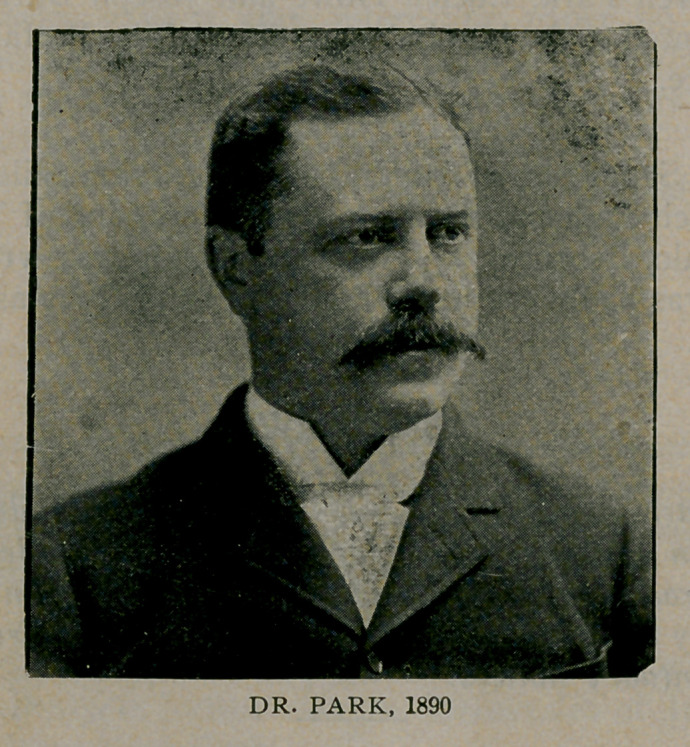


**Figure f2:**
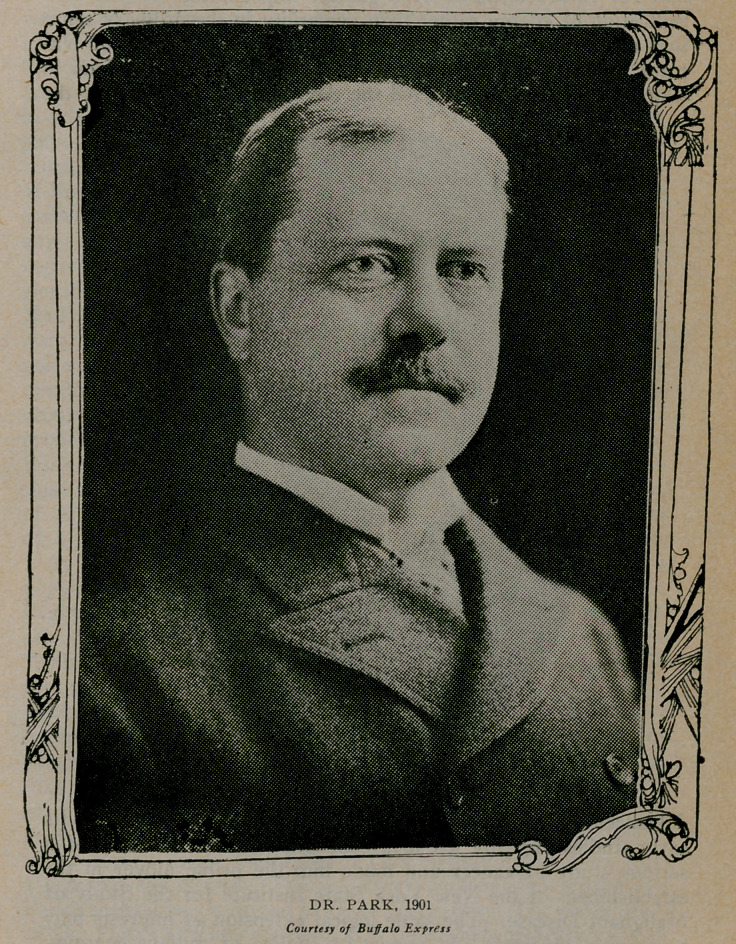


**Figure f3:**